# Investigation of antibacterial effect of Cadmium Oxide nanoparticles on *Staphylococcus Aureus* bacteria

**DOI:** 10.1186/s12951-014-0026-8

**Published:** 2014-07-25

**Authors:** Bahareh Salehi, Sedigheh Mehrabian, Mehdi Ahmadi

**Affiliations:** 1 Young Researchers and Elites Club, North Tehran Branch of Islamic Azad University, Tehran, Iran; 2 Microbiology Group, Biological Sciences Faculty, North Tehran Branch of Islamic Azad University, Tehran, Iran; 3 Institute of Biochemistry and Biophysics, University of Tehran, Tehran, Iran

**Keywords:** Antibacterial effect, Cadmium Oxide nanoparticles, Staphylococcus Aureus, Environmental factors

## Abstract

**Background:**

Inorganic antibacterial factors provide high bacterial resistance and thermal stability. Inorganic nanomaterial consists of modern formulation, biological, chemical, and physical properties produced on the basis of their function and influenced by their nano scales, the reason for which they have become very popular. The antibacterial effect of Cadmium Oxide Nanoparticles on *Staphylococcus Aureus* has been studied for the first time in this research because of their resistance to antibiotics.

**Materials and methods:**

Different concentrations consist of 10 *μg/ml,* 15 *μg/ml, and* 20 *μg/ml* have been provided and their effects were studied in the agar and broth against the foregoing bacteria. Needless to say, the optimization of their non-microbial effect in variable times, pH, and temperatures of exposure was analyzed.

**Results:**

The results represented that there is a direct association between the nanoparticles applied dosage and the restrain effect augmentation of applied dosage results in increase in restrain effect. In the study of environmental factors (pH and temperature), the results are in line with the inherent physiology of the bacteria; however, there was a significant decline in the number of analyzed bacteria cells due to the “Double Effect” of nanoparticle-pH variations as well as nanoparticle-temperature variables. In the very study, the promotion of Cadmium Oxide nanoparticles concentration leads to the elevation of antimicrobial feature and the reduction of bacteria growth rate is consistent with the other surveys about the nanoparticles effects on microorganisms to be more specific, one can come to this conclusion that the presence of nanoparticles prompts cellular destruction.

**Conclusion:**

In the recent study, by elevation in Cadmium Oxide nanoparticles concentration, the antimicrobial property augments and the bacteria growth rate declines, that are in line with other researches about the nanoparticles effect on microorganisms.

## Background

Nanoparticles high potentials caused their application in variable and precise processes, more specifically, in the biology and pharmacology which has been noticed by many biologists. Recently, along with the raising importance of healthcare, a large number of researches have been conducted to improve the antibacterial feature of nanoparticles. However, the application of certain antimicrobial materials has been restricted due to their lesion or toxicity. Inorganic antibacterial factors have a very high bacterial resistance and thermal stability. In recent years, researchers have highly noticed the Cadmium Oxide, its applications and properties in optoelectronic devices such as: solar cells [1], optical transistors, glassy electrodes, gas sensors, etc. [2]. These applications of Cadmium Oxide have been resulted from its individual electrical and optical features. Cadmium Oxide nanoparticles have been applied by many scholars up to now. In the same way, we have used Cadmium Oxide nanoparticles to confront bacteria which are pathogenic [3],[4]. *Staphylococci* are Gram-positive sphere shaped cells that generally array in form of irregular groups like grape clusters and grow in many mediums as well. *Staphylococcus Aureus* produces variable toxins and enzymes which are the major reason of bacteria survival; proteins, fats, and carbohydrates breakdown in order to provide necessitate materials, resistance against drugs and the ability of bacteria to cause disease. Some of these enzymes are Coagulase, Hemolysin, Leukocidin, Penicillinase, Lipase, Hyaluronidase, Catalase, and Protease. The enterotoxins of this microbe are dispersed by bacteria cells into the food or medium. The enterotoxin producing *staphylococci* are always able to produce Coagulase, but not all the positive Coagulase *Staphylococci* are usually capable of producing enterotoxin [5]. The synthesis of the nanomaterial effective on bacteria with high efficiency can be applied for disinfection and the elimination of environmental and industrial bacteria. It is expected that nanomaterial obtained in a variety of synthesis procedures enjoying different properties; hence, its antibacterial effect is essential. As it is difficult for most people to cope with the rising cost of combating pathogenic bacteria, finding a low price and prompt method to control its development and activity is a matter of the utmost importance. According to the fact that the bacteria are more resistant to prevalent drugs, the use of nanoparticles in hygiene and medicine is putative and they can be appropriate alternatives for traditional antibiotics; moreover, the production cost is lower and their storage is much easier compared to any other medicine. *Staphylococcus Aureus* is one of hospital’s infectious resistant to traditional antibiotics, such as Beta-lactam, and is responsible for Gastroenteritis led by producing enterotoxin in food. Due to the importance of noted issues, in this research, we intend to study the effect of Cadmium oxide nanoparticles on *Staphylococcus Aureus.* We analyze the antibacterial effect of Cadmium Oxide nanoparticles on *Staphylococcus Aureus* bacteria in this study.

## Results and discussion

### Absorbance spectrums UV–Vis of Cadmium Oxide nanoparticles

**Figure 1:**
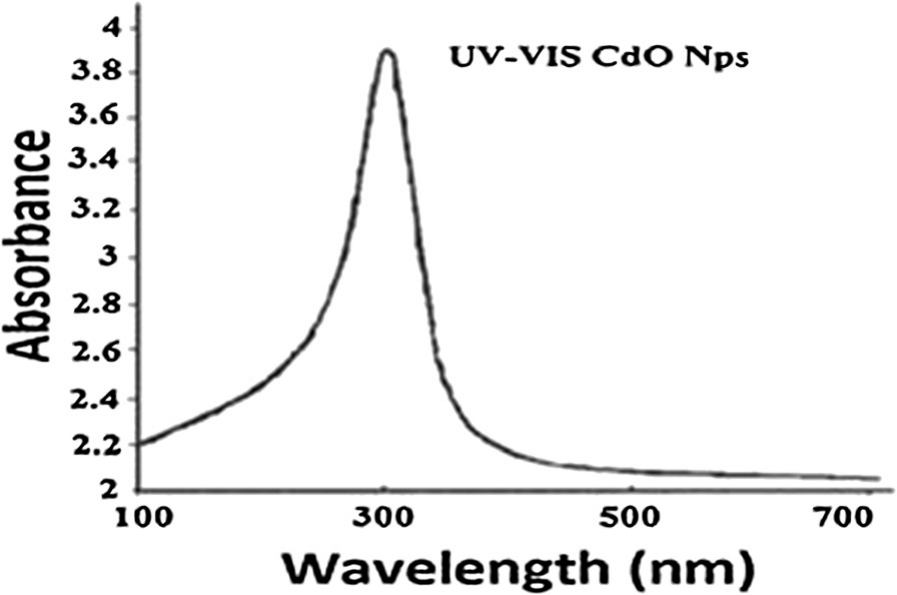
The UV absorbance spectrum for CdO nanoparticles.

This spectrometry is in regard to the transmissions between the electron scales. Generally, such transmissions are made between bonding orbital or non-bonding electron pairs and non-bonding orbital. Consequently, the link between the absorbance peaks wavelength and bonds emerged in the case study species seems to be feasible [6]. Visible-Ultraviolet spectrums of Cadmium Oxide nanoparticles are appeared in Figure 1. Although the wavelength of spectrum is limited by means of the light source, the absorbance band of nanoparticles represents a conversion in color location resulted from the amount of available limitation in the specimen comparing to the Cadmium Oxide nanoparticles. This optical phenomenon represents that these nanoparticles illustrate the level of quantum effects [7]. At the very level, the development of nanoparticles depends on the surfactant and organic solvent, since the Cetyl Trimethyl Ammonium Bromide (CTAB) surfactant helps to the cohesion of synthesized nanoparticles’ surface. Therefore, as a result of this interaction, stabilizing of particles and balancing the development or growth of the particles’ cores are emerged to achieve a high level of uniformity [8]. The Acetic acid and Ethanol solvent assist the dispersion of particles identically, a deliberate growth of particles in limited sizes, and the prevention of the particles integration [9].

### Electron microscope analysis

**Figure 2:**
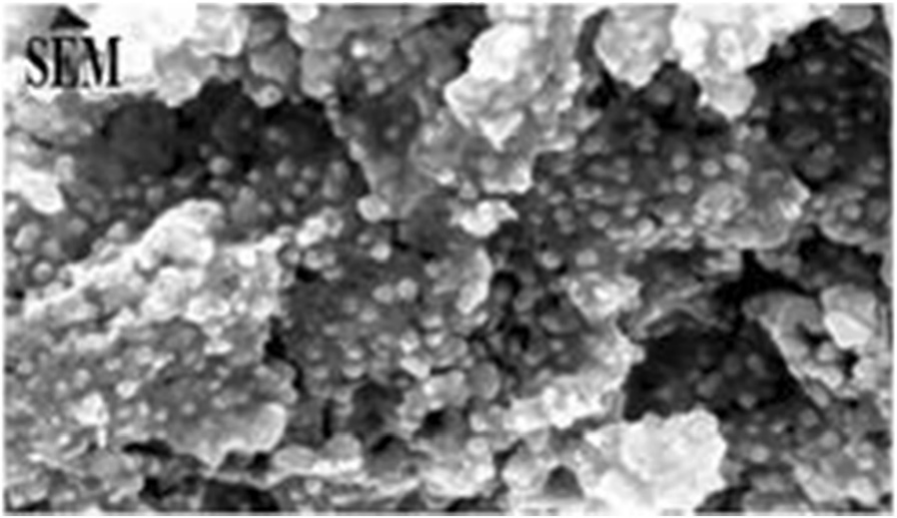
Image of electron microscope scanning of synthesized Cadmium Oxide nanoparticles with the magnification of 13000 times.

The image of synthesized Cadmium Oxide nanoparticles is visible in the Figure 2. This image is taken with the composite electron microscope with the magnification of 13000 times representing an approximate 30 nm diameter of synthesized nanoparticles.

### The analysis of inhibitory effect of Cadmium Oxide nanoparticles on *staphylococcus aureus* bacteria in the agar

The growth phenomenon and the activity of bacteria against variable concentrations of 10 μg/ml, 15 μg/ml, and 20 μg/ml of nanoparticles were analyzed. As it is obvious, the inhibition zone diameter which the *Staphylococcus Aureus* bacterium develops against the Cadmium Oxide nanoparticles indicates it could stop the growth of bacteria at a high level and there would be a direct relationship between the inhibitory effect and the nanoparticles applied dosage and it could be applied as a material including antibacterial properties (Table 1).

**Table 1:** Evaluation results of the inhibition zone diameter in variable concentrations of Cadmium Oxide nanoparticles on case study bacteria

**20 mg/ml**	**15 mg/ml**	**10 mg/ml**	**control**	**Concentration**
**19 ± 2 mm**	**14 ± 2 mm**	**10 ± 2 mm**	**0.0 mm**	***Staphylococcus Aureus***

### The analysis of Cadmium Oxide nanoparticles inhibitory effect on *staphylococcus aureus* bacteria in the broth

**Figure 3:**
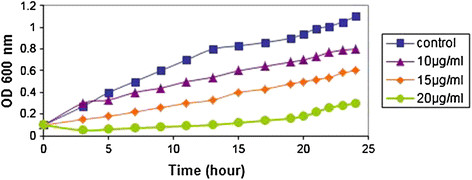
**The effect of variable Cadmium Oxide nanoparticles concentrations on** ***Staphylococcus Aureus*** **bacteria.**

To analyze the Cadmium Oxide nanoparticles inhibitory effect on *Staphylococcus Aureus* bacteria in the broth, different concentrations 10 μg/ml, 15 μg/ml, and 20 μg/ml of nanoparticles were applied. No nanoparticle was used for the control group. Therefore, the analysis carried out on the broth consists of four groups. The lids of the dishes containing treated broths and the control broth were shut tightly with the corks and they have been cultured to 37°C for 24 hours. Optical absorbance in the wavelength of 600 nm was utilized, the absorbance of the above solutions in Spectrophotometer was analyzed in order to accurately measure the bacteria concentration, and its diagram was drawn (Figure 3) for the purpose of analyzing the bacteria growth in every condition.

The 20 μg/ml concentration of Cadmium Oxide nanoparticles has provided the most inhibition effect on *Staphylococcus Aureus* bacteria. The effects of diverse Cadmium Oxide nanoparticles concentrations were studied on the number of *Staphylococcus Aureus* bacteria. The most inhibition effect has emerged on higher nanoparticles’ concentrations and the value of *OD* is significantly declined by the statistical aspect (P < 0.05).

### The analysis of inhibitory effect of Cadmium Oxide nanoparticles on the number of *staphylococcus aureus* bacteria

**Figure 4:**
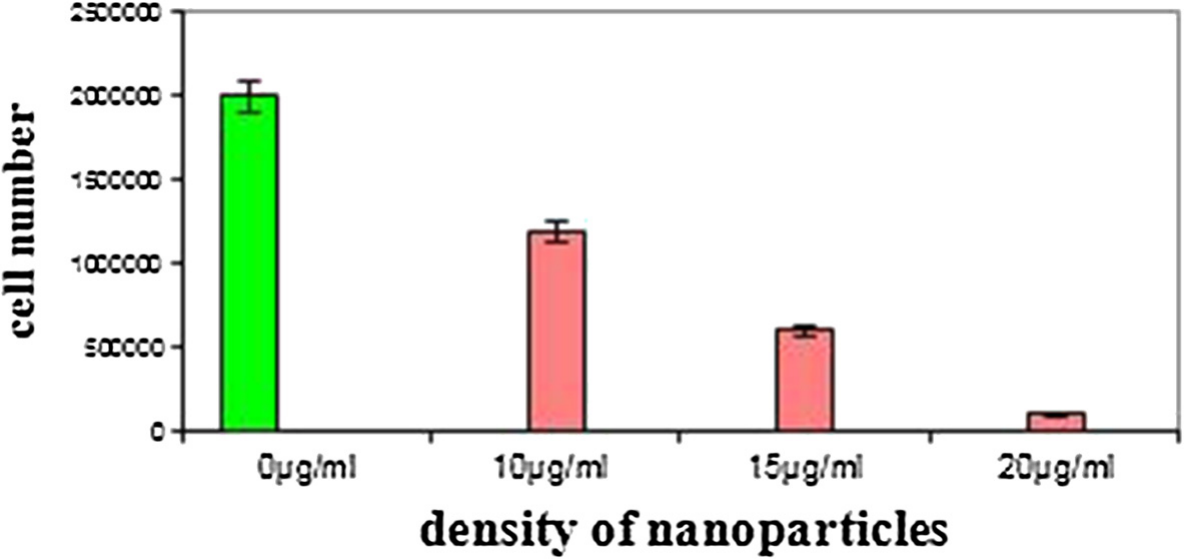
**The inhibitory effect of Cadmium Oxide nanoparticles on** ***Staphylococcus Aureus*** **Bacteria.**

The most inhibition effect (P < 0.05) with regard to 20 μg/ml concentration represents the antibacterial properties of Cadmium Oxide nanoparticles; the very property exceeds by an increase both in the concentration and in the maximum concentration made the bacteria to deteriorate to less than the 15% of original quantity (Table 2 and Figure 4).

**Table 2:** Analysis results relevant to the effect of Cadmium Oxide nanoparticles concentration on the number of case study bacteria

**20 mg/ml**	**15 mg/ml**	**10 mg/ml**	**control**	**Concentration**
**100,000 ± 100**	**600,000 ± 100**	**1,200,000 ± 100**	**2,000,000 ± 100**	***Staphylococcus Aureus***

Along with the enhancement in the concentration of Cadmium Oxide nanoparticles, the number of bacteria is deteriorated. The number of cells has a direct relationship with the applied concentration of nanoparticles in *Staphylococcus Aureus* bacteria, and it can be concluded from the regression ratio output that this relationship is negative, i.e., by the increase in concentration, the number of cells descends, but the P value in less than 0.05 and it represents the significance of this relationship.

### The analysis of temperature on the *Staphylococcus Aureus* bacteria in broth

**Figure 5:**
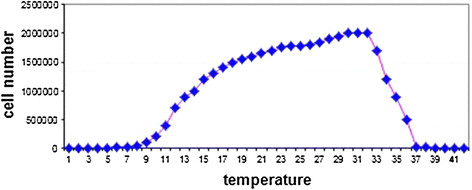
**The temperature effect on** ***Staphylococcus Aureus*** **bacteria in the presence of Cadmium Oxide nanoparticles.**

**Figure 6:**
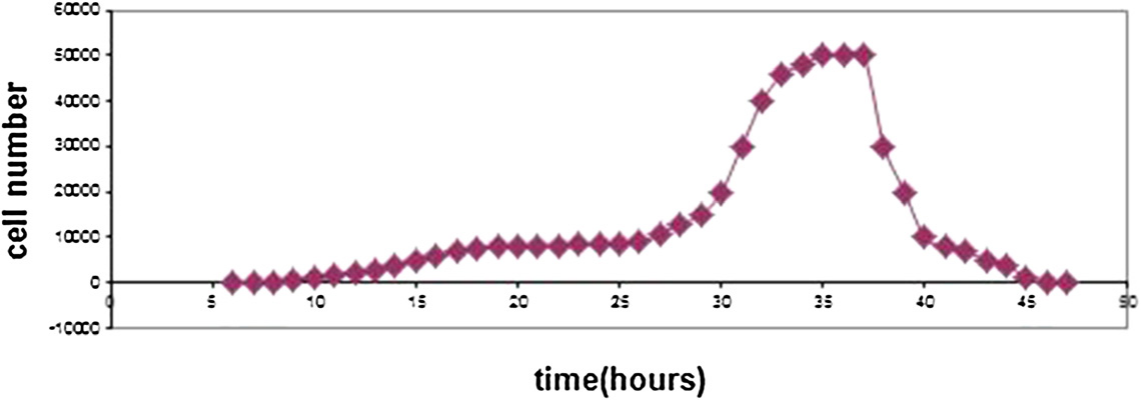
**The temperature effect on** ***Staphylococcus Aureus*** **bacteria in the broth.**

According to the given data from Figure 5, it is crystal clear that, the minimum temperature required for the *Staphylococcus Aureus* bacteria growth is 8°C and the maximum temperature for the growth of this bacterium is 45°C; moreover, the optimum temperature for this bacterium is between 35°C and 37°C. The achieved results have conformity with the bacterium physiology. The results of thermal effect for this bacterium show that these results are in line with the inherent physiology of this bacterium against the temperature. However, as it is clear, due to the Double Effect phenomenon of both nanoparticle and the temperature conversions, a significant deterioration (P < 0.05) was emerged in the number of bacteria cells (Figure 6).

### The pH effect analysis on *Staphylococcus Aureus* bacteria in the broth

**Figure 7:**
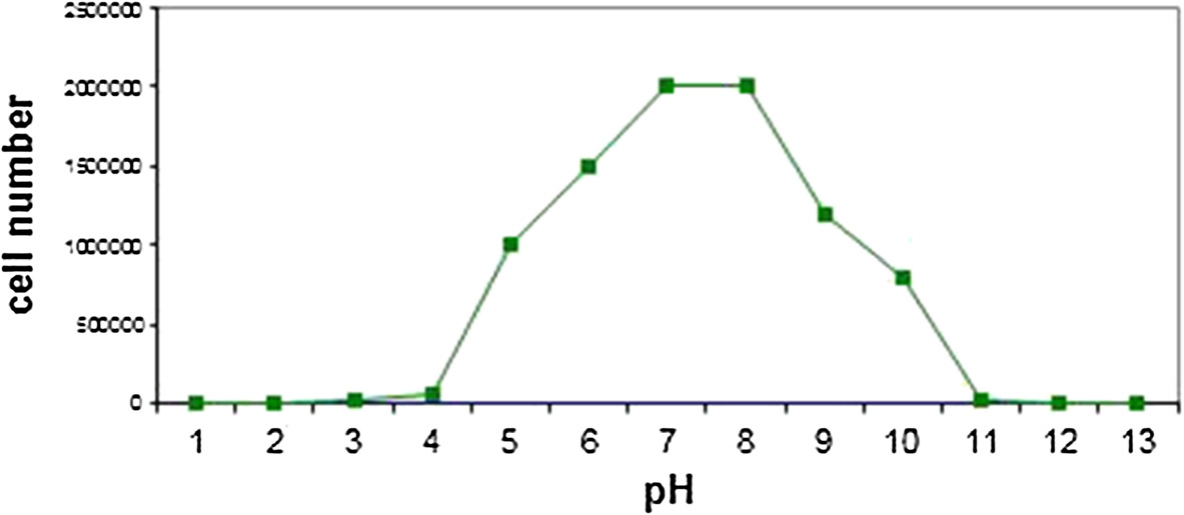
**pH effect on** ***Staphylococcus Aureus*** **bacteria in the broth.**

The pH effect on *Staphylococcus Aureus* bacteria in the absence of Cadmium Oxide nanoparticles and the presence of Cadmium Oxide nanoparticles was analyzed in this study and the results are demonstrated in Figures 7.

**Figure 8:**
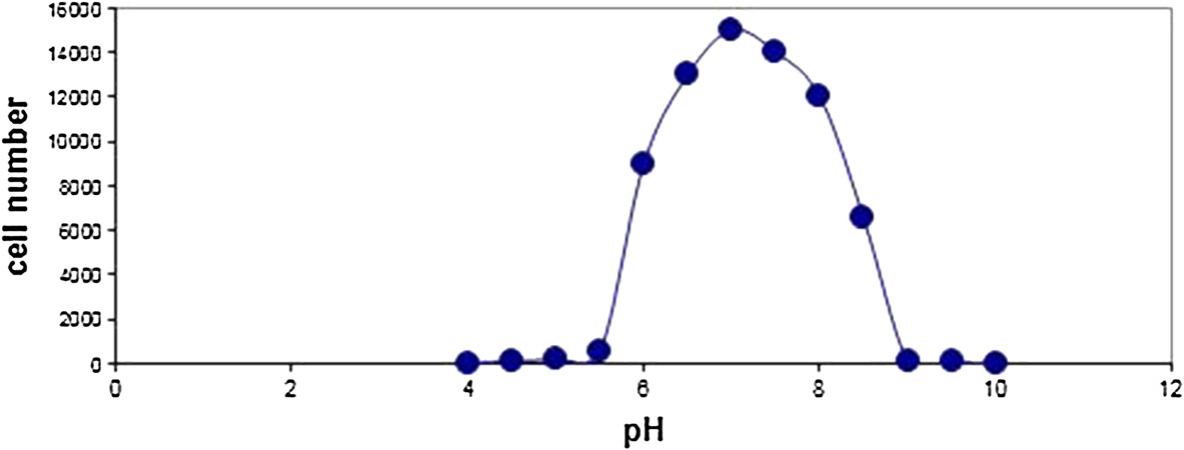
**The pH effect on** ***Staphylococcus Aureus*** **bacteria in the presence of Cadmium Oxide Nanoparticles.**

According to the data adopted from Figure 8, it is found that the minimum required pH for the growth of *Staphylococcus Aureus* bacteria is 4.5 and the maximum is 9.3, and the optimum pH is between 7 and 7.5. The obtained results ensure conformity with the physiology of the bacteria. These results are in line with the inherent physiology of the bacteria against pH. As it is obvious in Figure 8, as a result of Double Effect made by both of nanoparticle and the temperature conversions, a significant deterioration (P < 0.05) has been developed in the number of bacteria cells.

### Optimization of Cadmium Oxide nanoparticles antibacterial effect in variable times

**Figure 9:**
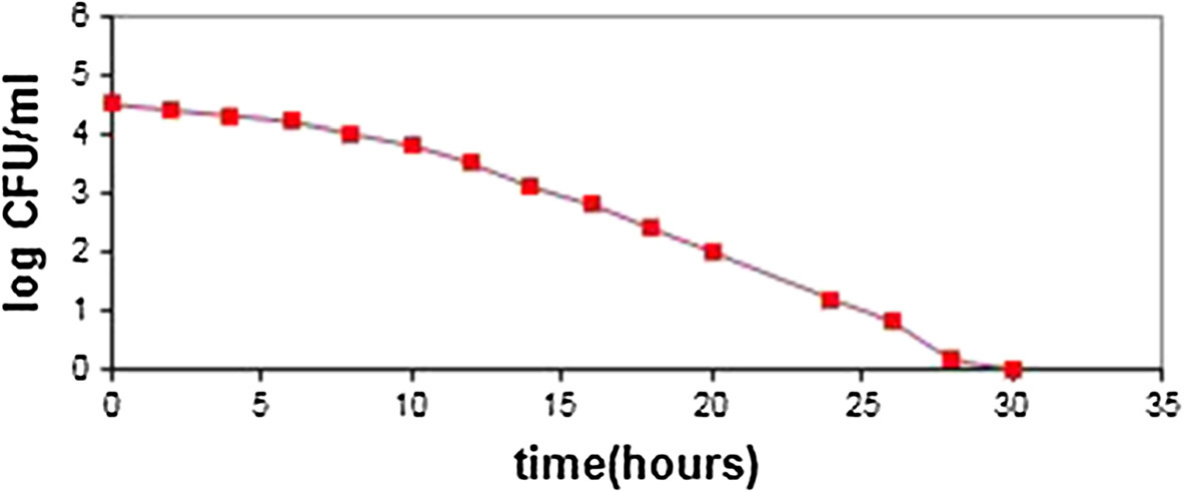
**The effect of maximum concentration (20 μg/ml) Cadmium Oxide nanoparticles on** ***Staphylococcus Aureus*** **bacteria viable cells.**

In the final analysis, the living cells of *Staphylococcus Aureus* bacteria were exposed to maximum concentration (20 μg/ml) of Cadmium Oxide nanoparticles in 37°C water. The results illustrate that in the control group, a decline in *Staphylococcus Aureus* bacteria concentrations reaches from 6.3 log CFU/ml to non-evaluable concentrations after 14 days. Neverthless, by adding nanoparticles to the broths of these bacteria, the viability of bacteria deteriorates from 14 days to less than two (Figure 9).

Through an elevation in concentration and time *OD* declines and there is a significant relationship between *OD* and time and concentration of applied nanoparticles in Dunnett test. Furthermore, in two-way *ANOVA* test, the identical assumption of *OD* value in different times and concentrations is rejected, because: (P < 0.05).

Nanomaterials have a huge number of functions in medicine; as antibiotics eradicate a little number of pathogenic factors, those are capable of about 650 types of pathogenic factors [10]–[12]. Cadmium oxide nanoparticles in 20 μg/ml concentration showed antibacterial effect on the resistant bacterium against antibiotics that was applied for this research. In research held by Buzby et al., they studied the antibacterial effects of Ag nanoparticles by employing them in 10-15 nm size and reported that an increase in nanoparticles effect depends on consumed values. [13]. It was found that by an increase in Cadmium oxide nanoparticles number, the antibacterial effect increases and the bacterium growth rate decreases. In another research by Sundrerajan, the antibacterial effects of MgO nanoparticles on gram positive *Staphylococcus Aureus* and gram negative *E.Coli* were studied and it was found that the inhibition zone for gram positive bacterium had been greater than gram negative one [14]. In the other research, the researchers studied antimicrobial effects of CeO_2_ nanoparticles on *Staphylococcus Aureus*. Their applied nanoparticles size was 37.6 nm and their results showed that the antibacterial effects of CeO_2_ depend on applied doses and the inhibition property developed by nanoparticles attested their antibacterial effect [15]. In 2009, Ayala et al. could inhibit *Staphylococcus Aureus* by Ag nanoparticles. They could also verify the antibacterial effect of Ag nanoparticles by well diffusion method protocol, subsequently, they could define minimum inhibitory concentration (MIC) of which by macro dilution method [16]. In research by Rafie et al. [17] in Egypt, they could control *Staphylococcus Aureus* and *E.Coli* by using Ag nanoparticles inside cotton crop. In a research, the researchers studied the effect of Titanium Oxide nanoparticles on *Staphylococcus* and they concluded that those have descent antibacterial effect on this gram positive bacteria [18] of which the same results of present study have achieved in antibacterial effect. Experiments were done to study the CrO nanoparticles toxicity on gram positive bacteria of human immune system and gram negative bacteria such as *Shigella* that their results imply the toxic nature of CrO nanoparticle for variable microbial systems and human T-lymphocytes [19]. In 2011, researchers studied CrO and CoFe_2_O_4_ nanoparticles impacts on *Staphylococcus* and the results showed that CrO has higher bacterial killing power against *Staphylococcus Aureus* in comparison with CoFe_2_O_4_ and in general, both nanoparticles have antibacterial effect; however, CrO had a better function [20] showing what we approved in the antibacterial effect of Cadmium oxide nanoparticles in this research. As for the high toxicity of Cadmium oxide nanoparticles, they are used to eliminate *Staphylococcus Aureus* in vitro and to eradicate the environment bacteria as well as cleaning medical supplies and equipment contaminated by that bacteria.

## Conclusion

In the recent study, along with the elevation in Cadmium Oxide nanoparticles concentration, the antimicrobial property augments and the bacteria growth rate declines being in line with the other researches about the nanoparticles effect on microorganisms. It can be concluded that at the presence of nanoparticles the cell destruction prompts. It is proposed to use cadmium oxide nanoparticles in elimination of environmental bacteria resistant to traditional antibiotics.

## Methods

Two different methods were used to analyze the sensibility of *Staphylococcus Aureus* to Cadmium Oxide nanoparticles in order to confirm the results. Different concentrations of each nanoparticle were separately cultured on *Staphylococcus Aureus* (according to half McFarland) in the Mueller Hinton Agar bearing different Cadmium Oxide nanoparticles. No nanoparticles medium was used for control group. The bacteria grew in 37°C for 24 hours. Then, the number of colonies was compared with the specimen, and to enumerate a hemocytometer, a scaled lam with definite volume was used. Subsequently, the number of bacteria in the spaces of lam was enumerated, next, 9 ml of the medium suspension was mixed with 1 ml of methylene blue and it was found that the bacteria took the color and converted to blue. In the second method, the bacteria in Trypticase™ Soy Broth were placed separately. The number of bacteria was measured every one hour. Optical density (*OD*) in the wavelength of 600 nm was applied by the absorbance of foregoing solutions in the spectrophotometric device to measure the concentration of bacteria. The number of bacteria was compared with the control group in variable times and turbidimetric analysis was carried out for enumerating the bacteria in the medium. In this method, some of the bacteria were posed in cultured suspension in a test tube with definite diameter and the tube in was placed the optical beam of spectrophotometer with 600 nm wavelength in a way that, initially, 100 ml of nutrient broth sterile was added to any of 11 sterile tubes and then 100 μl of nanomaterial suspension was added to the tube no. 1. After shaking the content of the tube, 1 ml of the material was transferred to the tube no. 2. In the same way, we provided the dilution series up to the tube no. 9 and shook the content of each tube subsequently. The tubes no. 10 and 11 were considered as the evidence. The tube no. 10 was considered as the evidence for nanoparticles suspension (containing bacteria and no nanoparticles) and the tube no. 11 as the evidence for the medium (with no bacteria cells and nanoparticles). By addition of 100 μl of the bacteria suspension to serial dilution tubes and heating the tubes to reach a concentration of half McFarland in 37°C, the minimum of growth inhibitory concentration was determined accurately for each bacterium.

### Preparation and the method of Cadmium Oxide nanoparticles analysis

To produce Cadmium Oxide nanoparticles in this study, in one experiment, the first solution sample with 0.06M Acetic acid and 0.03M Cadmium Sulfate was provided with 40 mg Cetyl Trimethyl Ammonium Bromide (CTAB) as the surfactant in 1 liter of double distilled water. Subsequently, the first solution was added to the second one and the obtained sediment was filtered by the Whatman filter paper, then, it was dried out in hot air stove of 80°C for 1 hour approximately. In the next level, it was transmitted to Silica crucible (41°C) and burned in 400°C about 2 hours, then, the resulted powder was cleansed with Ethanol to eliminate the impurities available in the particles for 3-4 times. The characteristics of obtained nanoparticles were studied by the use of X-ray diffraction, visible Spectroscopy absorbance - Ultraviolet, and it was utilized in order to study its antibacterial characteristics. Morphological analysis and the observation of synthesized Cadmium Oxide nanoparticles were carried out with the visible Spectroscopy-Ultraviolet device, double beam TU-1901, X-ray diffraction device D/Max-RA employing CuKα radiation and composite Electron microscope JEM-200CX [21].

### Selection, separation and cultivation of clinical specimens

To provide clinical specimens, the *Staphylococcus Aureus* bacteria were separated from urine culture of the hospital remedial ward.

### Provision of bacteria and cultures

*Staphylococcus Aureus* bacteria were provided from Shiraz University of Medical Sciences and approved by means of microbiological common methods. Broths and agars were purchased from the Merck Company of Germany.

### Preparation of Mueller Hinton agar

The Agar was provided in compliance with the instruction written on the package; 11.4 g of the powdered material for agar were solved in the distilled water under 25°C heat to reach the pH equal to 7.3 ± 0.2. Initially, the Agar was heated and corked firmly, and held on flame to make the culture identical, then, it was placed in autoclave to be sterilized in 121°C for 15 min. and put in refrigerator afterward. Subsequently, they were put in Petri dish; their lids were shut and kept reversely (to prevent vapor infiltration).

### Preparation of nutrient broth

The medium was prepared in compliance with the instruction written on the package. 0.65 g of the powder was taken and poured into the beaker with 50 ml of distilled water being stirred until it acquired the pH of 7.3 ± 0.2. This is a nutrient broth, poured into a flask and placed in autoclave. To remove the microbe from the broth and place it in the agar, a swab sterilized by the autoclave in 120°C for 15 min was used, then, they are kept in the refrigerator up to their time-of-use.

### The culture of bacteria and study of Cadmium Oxide nanoparticles effect in agar

Initially, four holes were emerged in specific points of the agar to hold three of Cadmium Oxide nanoparticles and the fourth for the control group (at the middle of agar). Subsequently, the sterile swab was entered in nutrient broths which *Staphylococcus Aureus* bacteria were grown in and was previously provided, and smeared it with the bacteria, then it was spread in the agar plates in three dimensions to cover the plate surface wholly. Later on, we selected the foregoing nanoparticles’ solutions provided with the mean and steady concentration of 15 μg/ml amongst the three concentrations of 10 μg/ml, 15 μg/ml, and 20 μg/ml for the purpose of standardizing, and infused into the determined shaft, and in the mid shaft, we infused the control solution which is distilled water in this study. Afterwards, the plates were placed into the incubator in 37°C for 24 hours and observed the effect of nanoparticles on the growth of bacteria, and finally, the colonies of bacteria were enumerated.

### Preparation of TSB (Trypticase™ Soy Broth), culture of bacteria and analysis of Cadmium Oxide nanoparticles effect in broth

The broth was prepared to comply with the instruction written on the package. 3 g of the powder was taken and infused into the beaker with 100 ml distilled water of which a pH of 7.3 ± 0.2 was acquired, and finally the solution poured and divided into eight test tubes. Then, we sterilized them in the Autoclave. We cultured the *Staphylococcus Aureus* bacteria in four separate tubes and considered one of these four as the control group. Consequently, we added every three different concentrations of Cadmium Oxide nanoparticles for each bacterium in three reminder tubes complying with half McFarland and shut the lids of containers holding treated broths (bacteria + Cadmium Oxide nanoparticles); the control broth was shut tightly with a cork, shook aerobically, and placed in the incubator in 37°C for 24 hours. Afterwards, the optical density with the wavelength of 600 nm was used applying the foregoing solutions absorbance in Spectrophotometer to measure the concentration of the bacteria and its diagram was drawn so that, we could study the growth of bacteria in any situation. The antimicrobial effect of Cadmium Oxide nanoparticles in broth was analyzed, as their control group was distilled water. Their absorbance was read with the Spectrophotometer every one hour and their absorbance diagram in time unit were drawn for the specimens. All of obtained conclusions in tests were compared with the control group.

### Statistical analysis

Statistically, all the results were shown as the average and deviation forms. After determining the data distribution, to compare the results of every value in each of the groups before and after the study, the ANOVA test with continuous measurement and to compare the groups the one-way ANOVA, *Dunnett* test, and regression were used. Moreover, a significant level less than 0.05 was assigned for all the analyses.

## Abbreviations

TEM: Transmission electron microscopy

UV-Vis: Visible-Ultraviolet spectrums

OD: Optical density

CTAB: Cetyl Trimethyl Ammonium Bromide surfactant

## Competing interests

The authors declare that they have no competing interests.

## Authors’ contributions

BS conceived the project, and revised the manuscript and the biological experiment and performed the experiments of nanoparticle assembly and *in vitro* test. SM designed the experiment. MA has done statistical analysis. All authors read and approved the final manuscript.
